# The Development and Growth of the English National Real-Time Syndromic Surveillance Program: Key Developments and Lessons Learned From the First Two Decades

**DOI:** 10.2196/73373

**Published:** 2025-09-19

**Authors:** Gillian E Smith, Natalia R Jones, Sally E Harcourt, Sue Smith, Paul Loveridge, Roger A Morbey, Helen E Hughes, Obaghe Edeghere, Daniel Todkill, Sue Ibbotson, Duncan Cooper, Brian McCloskey, Alex J Elliot

**Affiliations:** 1 Real-time Syndromic Surveillance Team, Chief Medical Advisor Group, UK Health Security Agency, 23 Stephenson Street, Birmingham, B2 4BH, United Kingdom, 41 1212329211; 2 National Institute for Health and Care Research Health Protection Research Unit (NIHR HPRU) in Emergency Preparedness and Response, King's College London, London, United Kingdom; 3 School of Environmental Sciences, University of East Anglia, Norwich, United Kingdom; 4 Health Equity and Clinical Governance, Chief Medical Advisor Group, UK Health Security Agency, London, United Kingdom; 5 NHS Bradford Districts Clinical Commissioning Group, Bradford, United Kingdom; 6 Department of Infectious Disease Epidemiology and International Health, London School of Hygiene and Tropical Medicine, London, United Kingdom

**Keywords:** syndromic surveillance, epidemiology, England, health protection, infectious disease, all hazard

## Abstract

Syndromic surveillance now forms an integral part of the surveillance for a wide range of hazards in many countries. Establishing syndromic surveillance systems can be difficult due to the many different sources of data that can be used, cost pressures, the importance of data security, and the presence of different (and rapidly evolving) technologies. Here we describe major points in the development of the UK Health Security Agency English real-time syndromic surveillance service over its first 2 decades (1998 to 2018). We identify the key wider themes that we believe are important in ensuring a sustainable and useful syndromic surveillance service. We conducted semistructured interviews with current members of the UK Health Security Agency syndromic surveillance team who were involved from the earliest stages and previous senior colleagues who were supportive of the syndromic surveillance work during the early phases. For this viewpoint, we partitioned the development of syndromic surveillance in England into 3 time periods: 1998 to 2005 (“the beginnings”); 2006 to 2011 (“the growth phase”); and 2012 to 2018 (“mainstream”). We asked the interviewees for their views about the development of syndromic surveillance, and in particular the main drivers and events, the team and system, and outputs and uses. The results from the interviews highlighted some key themes including the integration of syndromic surveillance into the public health system, creativity, good collaboration and teamwork, leadership and determination to persevere, and agility and the ability to adapt to new threats. Using the results of the discussions and our personal experience of running the syndromic surveillance service from inception and over decades, we constructed a set of recommendations for establishing and running sustainable syndromic surveillance systems. In this age of increased automation, with the ability to transfer data in real-time and to use machine learning and artificial intelligence, we are approaching a “new age of syndromic surveillance.” We consider that the focus on the public health questions, relationships, collaboration, leadership, and true teamwork should not be underestimated in the success of and usefulness of real-time syndromic surveillance systems.

## Background

Syndromic surveillance provides early warning of public health incidents, situational awareness of ongoing incidents, and reassurance during large-scale events [1]. Used globally, syndromic surveillance uses real-time, automated data captured from sources such as general practitioners (GPs), emergency departments (EDs), telephone health helplines and websites, and pharmacists [2]. This allows for the early identification, distribution, and influence of public health hazards to be determined [3]. Although syndromic surveillance now forms an integral part of the surveillance of a wide range of hazards in many countries, establishing syndromic surveillance systems can be difficult because of the numerous different sources of data available, cost pressures, the importance of data security, and different technologies.

Syndromic surveillance in England developed from a small pilot project in 1998 using telehealth call data based in the West Midlands (a region of England) to a suite of systems used nationwide and constituting a key element of surveillance for communicable and noncommunicable diseases [4]. The UK Health Security Agency (UKHSA) national real-time syndromic surveillance program now consists of 6 national systems, each monitoring an anonymized feed of health data obtained from a different part of the English National Health Service (NHS). Daily feeds include NHS 111 calls (telehealth), NHS 111 online assessments, ED attendances, GP in-hours consultations, GP out-of-hours contacts, and ambulance dispatch calls. Daily anonymized data are received from each system, then analyzed, interpreted, and assessed, and, where necessary, action is taken [5]. Analysis and assessment include epidemiological, statistical, and risk assessment [6,7]. The team running the surveillance (the Real-time Syndromic Surveillance Team [ReSST]) is a multidisciplinary team of consultants, medical epidemiologists, epidemiological scientists, experts in informatics and database management, and statisticians (approximately 10 people).

Syndromic data are held securely in UKHSA databases, with processed, aggregated outputs made publicly available through routine reporting [8,9]. The role of the UKHSA syndromic surveillance program is to provide early warning of potential threats (eg, seasonal norovirus and pandemic influenza), situational awareness (eg, during a seasonal influenza epidemic), and reassurance of a lack of impact (eg, during a mass gathering). Syndromic surveillance supports and augments other national, regional, and local surveillance and health protection programs in UKHSA.

Elliot et al [4] recently described the evolution of technical and data aspects of this development, but there is little in the literature describing the process of development and the lessons learned for those developing syndromic surveillance across the world. Here, we describe major points in the development of the UKHSA English real-time syndromic surveillance service over its first 2 decades (1998 to 2018), and key, wider themes we believe are important in ensuring a sustainable and useful syndromic surveillance service. We draw upon over a century’s collective experience from the England syndromic team members and from key public health stakeholders who “championed” the development in the early stages.

For this viewpoint we conducted semistructured interviews with members of the UKHSA syndromic surveillance team who were involved in establishing and developing syndromic surveillance in England from an early stage. Participant selection was based on a convenience sample of past and current members of ReSST. In addition, we spoke to senior public health professionals who were supportive of the syndromic surveillance work during the early phases (termed “syndromic champions”). We conducted informal semistructured discussions with participants between October 2023 and April 2024, with 10 semistructured discussions undertaken in total (3 team members, 2 current or past team managers, and 5 syndromic champions). All discussions were conducted by GES using a semistructured questionnaire via videoconferencing (Multimedia Appendix 1). The discussions were informal, between colleagues or ex-colleagues (some of whom are authors of this paper), and consent was given for the discussions to be recorded. The discussions explored the reasons behind the sustainable development of the surveillance service, alongside the drivers of the development and significant events or outputs.

GES initially reviewed the transcripts and, using thematic coding, extracted key themes that were important in the development of the surveillance service. After discussion with NRJ, key themes were refined and developed into the discussion reported here. This was also supported and supplemented through the collective experience of GES and AJE, who have led the development of syndromic surveillance in England since its inception. In addition, GES reviewed key documents of the team, including notes of strategic meetings, reports on the work, and surveillance outputs (both routine and in support of incidents) between 1998 and 2018.

We divided the evolution of syndromic surveillance in England into 3 time periods, named according to the phases in syndromic surveillance development. The phases were 1998 to 2005 (“the beginnings”), 2006 to 2011 (“the growth phase”), and 2012 to 2018 (“mainstream”). For each of these phases, we considered 3 areas of development: the main drivers and events, the team and system, and outputs and uses.

## Summary of the Main Drivers, Team and System, and Outputs and Uses

The key events in the development of the syndromic surveillance service are summarized below (Multimedia Appendix 2).

### Main Drivers

During “the beginnings” (1998‐2005), key drivers of the development of syndromic surveillance in England included concern about the early detection of influenza outbreaks, and fears over biological terrorism, driven in part by the deliberate release of anthrax in the United States in 2001 [10-12]. Between 2006 and 2011 (“the growth phase”), factors driving the further development of syndromic surveillance included environmental incidents, such as the aftermath of the 2003 European heatwave [13] and the 2010 Icelandic volcanic ash plume [14], alongside the need to expand and further develop the service to support surveillance of mass gatherings, especially the London 2012 Olympic and Paralympic Games [15,16]. Once syndromic surveillance had become “mainstream” (2012‐2018), the development of the surveillance continued to be driven by both acute infectious and noninfectious incidents [17,18], alongside the desire to support research, for example, on the impact of vaccines [19,20], and the understanding of the value in early warning [21].

### Team and System

In “the beginnings,” there was little designated support to the team, and much of the work focused on collaboration with the original telehealth data provider and relied on communication via “fax” of small amounts of data. In “the growth phase,” the systems expanded considerably with the inclusion of GP in-hours and out-of-hours data and an ED system. The automation of the systems improved, and staffing in ReSST was expanded, in large part because of additional funding supporting the London 2012 Olympic and Paralympic Games. During the “mainstream” phase, statistical methodologies were refined to support the increasing amount of data and the scope of the systems, including the use of ambulance data and online data.

### Outputs and Uses

Throughout “the beginnings,” the surveillance was used to herald the rise of influenza in the community [10,22] and to provide real-time intelligence on presenting symptoms during events such as the Buncefield Fire [23]. Within “the growth phase,” the systems were used in an increasing scope of infectious (such as the 2009 H1N1 influenza pandemic [24]) and noninfectious (such as assessing the impact of heatwaves [13]) incidents. In the “mainstream” phase, the surveillance was considered to be “all hazard” and increasingly used to support a variety of infection and environmental incidents [17,18].

## Key Themes for the Success and Growth of the Service

The qualitative analysis of the information captured from interviews and discussions revealed the emergence of 5 key themes.

### Theme 1: Integration Into the Public Health System

Many interviewees commented on the focus of the surveillance being primarily and fundamentally practical public health and that the team was grounded in a public health organization:

… it was always informed by a real solid understanding and involvement, particularly through you [GES] in that frontline Health Protection response.

… here we were sort of coming at it from a real pure public health perspective.

In addition, the importance of the integration of surveillance into the wider public health system was highlighted. The initial idea for using a new national telephone health service, NHS Direct, for syndromic surveillance [10] came from the Regional Director of Public Health in the West Midlands, with strong public health support from leaders in the communicable disease surveillance system:

… because they [public health colleagues] were very much what does this mean for public health.

… what’s the added value which I think was something that gave us a bit of an edge.

Others mentioned the multipurpose nature of the surveillance. Although originally used for influenza, syndromic surveillance was developed to be part of bioterrorist surveillance, infectious disease surveillance, environmental type hazards, and mass gatherings:

The ability of syndromic surveillance to respond to potential terrorism events was hugely important in providing a sense of security after the events of 9/11 - in that atmosphere of a national threat, I felt its [syndromic surveillance] future was safe.

Not underestimating the ‘power of being able to walk into a room and provide reassurance’ in for example the 2012 Olympics.

### Theme 2: Creativity and Ability to ‘Build on a Good Idea’

There was no formal syndromic surveillance program in England prior to 1998, and interviewees commented on the importance of being able to “take a good idea and run with it.” Starting very small, with minimal resources and as a pilot project [22], the originators were able to show benefit and then be creative in considering the public health benefit over multiple areas [4]:

A good idea is a good idea only when you know it’s a good idea.

There was something early on about just allowing it to kind of grow a bit.

You've got the creative people, but you’ve got to have people that will let them be creative as well.

During “the beginnings,” no other group in the United Kingdom was developing this form of surveillance, and few globally. This was at the time a “niche area” that was ripe for public health evaluation. The public health and epidemiological approach (and determination) ensured that the work was validated and expanded into other areas, and the team was given time and freedom to explore this novel approach (without undue bureaucracy). Being right at the beginning of this development of the system that eventually became syndromic surveillance was perceived as being exciting and groundbreaking.

It was really exciting, felt a little bit like we were at the forefront of public health surveillance, like globally as well.

There’s sort of a gradual progression to say move from being an interesting surveillance thing to being part of public health to being part of the response and to be focused on generating action.

### Theme 3: Collaboration and Teamwork

Most interviewees stressed the importance of good collaboration and teamwork. This applied within the public health organization hosting the syndromic surveillance, and also across the organizations involved with supplying data, for example, the Royal College of General Practitioners (RCGP) and the Royal College of Emergency Medicine (RCEM). Such multiagency and multidisciplinary collaborations were felt to be vital to the development and success of the systems. Although it was important to foster close links with technical colleagues and those responsible for transferring data, in the early stages, we felt the development of relationships with those with strategic leadership in organizations (and particularly those who understood the potential public health and clinical benefit) was crucial. Such senior syndromic champions in the organizations were able to effect changes and ensure the “technical” and data requirements were followed.

Following initial idea needed a senior person with understanding how it could be used strategically and operationally and to link up with all the other surveillance systems and partners in the field and across the NHS.

So not just the quality of the relationships, but how do you work in partnership.

Interviewees stressed the importance of personal connections and people “getting” what we were trying to do. Colleagues valued the time spent working through what was trying to be achieved from a public health perspective in advance of the systems being used in a live public health event. Colleagues stressed the importance of these networks and personal connections and the importance of not neglecting these relationships or letting them “lie fallow.”

And the networks and the connections, then they can, you know, facilitate things happening…

For those developing the surveillance, we very much valued working with colleagues who had a different focus or priority and who were able to look at our work with a different “lens.” Clinical colleagues working on the front line also provided essential knowledge (eg, about what diagnostic codes might be used by clinicians for evolving conditions) and were able to challenge our assumptions about the interpretation of the data. Collaborating colleagues in front-line health care services were also crucial for shaping the surveillance outputs to ensure that they not only met public health and epidemiological needs, but also the needs of health services.

You do need people to sort of wave a flag for things to get going. It has been my experience.

If it hadn’t actually proved to be useful it those people wouldn’t have been able to continue to support it in the way.

Because of champions like yourself [ReSST] and others in other organisations that enable this to happen, because I think without that it would just become a data thing or you know.

From early stages, we established system steering groups to guide the work with syndromic champions across clinical organizations, public health, and academia. However, the leadership for the surveillance was embedded in a health protection organization.

I learned a lot from you [GES] in terms of bringing steering groups together of people with different skills that prepare to challenge, not necessarily people that were gonna agree with you.

… set up quite a broad thinking group think that that’s really, really important as well.

… ability to work with other people and other disciplines…..

### Theme 4: Leadership, Determination, and Perseverance

Several interviewees mentioned the importance of determination and perseverance in the initial work needed to get the syndromic surveillance accepted as part of wider surveillance, for example, as part of the surveillance program for influenza. Although the use of syndromic surveillance is now widely accepted as part of the surveillance “jigsaw,” this was not the case in the early stages. We received a challenge and “pushback” from those who felt that microbiological confirmations were needed for surveillance and that “clinical” and symptom-based surveillance had little to offer. We needed to both validate and conduct the research to demonstrate its worth—but also the leadership and determination to do so.

… have done such a great job over the years – the expertise of course, but also a dogged dedication and perseverance in the face of (1 or 2!) adversities and the development of a fab team that has offered such continuity – to have retained team members over such a long period of time must be a real rarity and is certainly a great achievement underpinning success.

This [establishing the syndromic surveillance systems] was not a walk in the park.

There were lots of other things that you could have done that would have been an awful lot easier.

… leadership and continuity and determination and drive.

One further theme that emerged was the longevity of the team members. Although the team started very small and there was no dedicated support other than a part-time epidemiologist, as the team started to grow, many members stayed in their roles for many years (several over 20 years). This team stability was a feature noted by several interviewees, and although the reasons may be varied, we think the novelty and constant evolution of the syndromic systems (and thus interest), the ability to support multiple types of events, the mutual support and “feeling of belonging,” and being involved in a unique surveillance area were important.

… and you know, I mean the team has been together for a remarkable length of time, 20 years.

… leadership and continuity and determination and drive and the fact that you've you know you’ve had a team who've been so focused and dedicated for such a long time because this really wasn’t an easy project.

… the development of a fab team that has offered such continuity – to have retained team members over such a long period of time must be a real rarity and is certainly a great achievement underpinning success.

### Theme 5: Agility and Adaptability to New and Emerging Threats

Interviewees commented on the importance of being “nimble” and adaptable to emerging threats. Although the initial work was focused on the surveillance of influenza-like illness, it rapidly became clear that syndromic surveillance is “multipurpose” and could be used not only for a variety of infectious diseases (such as respiratory or gastrointestinal infections), but also for environmental hazards (such as the impact of heatwaves).

We’ve learned how to use it, how to, and you know when, when it worked, when it’s best to use and I mean in some situations, obviously when the numbers are too small or we haven’t got representative data or whatever, it doesn’t work.

The big events that have been drivers that have pushed us up pushed us into the spotlight.

… as much politics as epidemiology.

We needed to be aware of the ongoing and potential public health threats and flexible enough to be able to use the data to support a variety of public health events, such as climate change–related, emerging infections, and so on.

You've been able to develop and exploit its potential to a significant extent.

… enable us to sort of say quite firmly I think what we could and what we couldn’t and sort of detect really and what the added value was.

Throughout the development, we were able to “piggyback” on NHS systems that were developed to manage clinical care. We used only anonymous data that were generated as part of a patient’s interaction with health care services. We were careful not to ask anything additional of those working in clinical care, but rather to use (with appropriate information governance procedures in place) the routinely generated data. This ensured that additional time was not needed for those working clinically and also kept costs to a minimum. Although the syndromic data were not “bespoke” for each event, we gained increasing familiarity about where syndromic surveillance could be useful and where it was unlikely to add to existing surveillance systems (eg, local small gastrointestinal outbreaks). Throughout the development, we tried to be as clear as we could about what could and could not be detected using syndromic surveillance, and our evidence for this evolved as the systems developed. We felt it was important to retain our focus and efforts on those events for which we believed that we could add value, for example, a generalized rise in vomiting suggestive of community norovirus activity rather than a small, localized rise in infections that may present in a variety of ways clinically.

## Key learning

Using semistructured discussions and a qualitative analysis, here we provide feedback and views from key experts and stakeholders who were integral to the initial concept and further development of the national syndromic surveillance program in England. We summarize the key developments in the surveillance service and, using the results of the discussions, draw out the views of respondents on the factors they considered important in enabling the syndromic surveillance to develop, grow, and become a mainstream component of surveillance for health protection in England today.

This feedback provides valuable lessons for the successful development and deployment of syndromic surveillance systems, but can also be applied outside of the field of syndromic surveillance. The interviews focused on understanding the reasons why the national syndromic surveillance program was successful. Key themes emerging included the importance of: integration into the public health system; creativity and ability to “build on a good idea,” leadership, determination and “doggedness,” and being agile and adaptable to new and emerging threats. These themes particularly revolved around people rather than technology or data [25]. This is an interesting result, as often surveillance is focused on developing technical solutions to extract, transfer, and analyze data. However, without the human skills and input required to initially identify and develop collaborations and working relationships with data providers to access data, the most sophisticated technological solutions would be fruitless.

One of the key strengths of our work is the longevity and rich history of the UKHSA syndromic surveillance team. At the time of the data collection, 5 internal interviewees had been in the UKHSA syndromic team for over 20 years each and were therefore able to provide an excellent retrospective record of the development of the team and systems over the last 2 decades. There are, however, some limitations to our approach. The nature of this work (describing over 20 years of development) introduces retrospective recall bias. It is difficult to quantify this bias; however, by interviewing several colleagues, we hoped to reduce that level of bias. Furthermore, we selected a convenience sample of key interviewees based upon their historical relationship with the team, which was entirely of a supportive nature. The style of this viewpoint meant it was appropriate that we spoke to those who were involved in the process. Although it is entirely possible that there are other colleagues who were not so supportive of syndromic surveillance, without current working relationships, it was not possible to select them for interview. Finally, while we feel that the results of this viewpoint are applicable internationally to other teams either planning the development of syndromic surveillance systems or more experienced teams looking to further develop their setup, the results are specific to the English public health and NHS, which we have to accept limits the overall generalizability outside of the United Kingdom.

In this age of increased automation, with the ability to transfer data in real-time and to use machine learning and artificial intelligence, we are approaching a “new age of syndromic surveillance” [4]. Now, much larger volumes of clinical data can be contemporaneously analyzed, and linkage can be made between individual symptoms, signs, and investigations (or test) results of the patient. These increasingly refined analyses may enable the identification, using syndromic surveillance, of rarer but more serious emerging illnesses in real time (eg, Shiga toxin–producing *Escherichia coli* infections and encephalitis).

## Recommendations

Our “look back” at the history of the development of syndromic surveillance in England suggests that the importance of focus on the public health questions, relationships and collaboration, leadership, and true teamwork should not be underestimated in the success of and usefulness of real-time syndromic surveillance systems. Using the results of the discussions and our personal experience of running the syndromic surveillance service from inception and over decades, we summarize our recommendations for establishing and running sustainable syndromic surveillance systems (Textbox 1), which augments our previous advice on the logistical and procedural steps required in setting up syndromic surveillance systems [1].

Textbox 1. **Key lessons from the development of real-time syndromic surveillance in England.**

- Focus on the public health questions and contribution of the syndromic surveillance (the data are important, but should follow the questions).
- Integrate the surveillance into a health protection–focused public health system.
- Get the “buy in” of senior public health colleagues (syndromic champions)—you will need their ongoing support to start and then develop the syndromic surveillance systems.
- Start simple and small and do not make it overly complicated—focus on one data source and develop strong and collaborative links with that data provider.
- Do not ask extra of busy front-line clinicians.
- The syndromic surveillance system is only as good as the team running it—invest in the people and the team, including their development.
- Aim eventually for a multidisciplinary team coordinating the syndromic surveillance system, including public health experts, epidemiologists, statisticians, informatics, and data scientists.
- All technology and methodologies have to be reliable and able to be executed each day by a range of skilled scientists and epidemiologists—do not make it overly complicated or it will not work.
- Be creative and opportunistic in the multiple uses of surveillance, that is, all hazard, supporting outbreaks, pandemics, mass gatherings, and noncommunicable diseases rather than solely focus on one disease area (eg, influenza).
- Look to the future—think “out of the box” about what future events could happen, how diseases might present, and what future technologies could be adopted.

## Supplementary material

10.2196/73373 Multimedia Appendix 1 Informal semistructured interview questions for the UK Health Security Agency syndromic “History” project: key events for syndromic surveillance in England over the first 20 years (1998-2018).

10.2196/73373 Multimedia Appendix 2 Examples of the main drivers, the important developments in the team and systems, and the main outputs and uses of the UK Health Security Agency syndromic surveillance service (1998-2018).

## Abbreviations

ED: emergency department
GP: general practitioner
NHS: National Health Service
NHS: National Health Service
ReSST: Real-time Syndromic Surveillance Team
UKHSA: United Kingdom Health Security Agency
