# Supplementary material for: The Development and Growth of the English National Real-Time Syndromic Surveillance Program: Key Developments and Lessons Learned From the First Two Decades
Source: J Med Internet Res. 2025 Sep 19;27:e73373. doi: 10.2196/73373 (PMC12448257; doi:10.2196/73373)
Supplement: Multimedia Appendix 1 [file jmir-v27-e73373-s001.docx]

**Informal semi–structured interview questions for the UKHSA syndromic ‘History’ project: key events for syndromic surveillance in England over the first 20 years (1998 to 2018)**

**Introduction**

Discuss why doing this project and what the proposed outputs are.

**What is the aim of this project?**

To document (in an informal and accessible way) the key events, decisions and personalities involved in the history of UK Health Security Agency (UKHSA) real-time syndromic surveillance over 20 years, particularly focussing on the early development.

**Why is it important?**

- UKHSA and its predecessor organisations have been at the forefront of the development of syndromic surveillance in England for over 20 years;
- Several of the people involved in the very early stages of syndromic stages are now retired or will retire in the next few years and there is a potential for this ‘history’ to be lost to the organisation and syndromic surveillance team;
- There is no record of the key dates in the development of national syndromic surveillance in England, or of the ‘softer’ information around the drivers for the surveillance and the key people involved and decisions taken.

**Questions**

What are your earliest memories of syndromic surveillance?

Prompt: Events/ people?

What were the key drivers in the initial stages?

Prompt: What do you think was the main reason for starting this process?

What existed before?

Who were the main supporters?

Prompt: People in UKHSA predecessor organisations / outside

Are there any key people /organisations without which it wouldn’t have happened?

What were the key challenges?

How did you overcome these?

What are the things you would include on a timeline (especially focussing on the initial stages) – provide draft timeline?

Is there any anecdote / key bit of information you would like to include? Have you got any photos / other memorabilia that would be interesting?
