# Supplementary material for: The Development and Growth of the English National Real-Time Syndromic Surveillance Program: Key Developments and Lessons Learned From the First Two Decades
Source: J Med Internet Res. 2025 Sep 19;27:e73373. doi: 10.2196/73373 (PMC12448257; doi:10.2196/73373)
Supplement: Multimedia Appendix 2 [file jmir-v27-e73373-s002.docx]

**Table S1.** Examples of the **main drivers for the development** of the UK Health Security Agency syndromic surveillance service (1998 to 2018).

| **‘The beginnings’: 1998 to 2005** |
| --- |
| 1998: Initial driver was for influenza surveillance and to detect influenza early.  Original idea and support for concept was by the then West Midlands RDPH – supported by Regional Epidemiologist, Director and Deputy Director of national Communicable Disease Surveillance and Control department for England.  2001: ‘The deliberate release of anthrax in the United States in autumn 2001, with heightened fears over the possibility of biological terrorism, led to joint project between the Communicable Disease Surveillance and Control department for England and NHS Direct. Aim of the project was to detect local or national increases in symptoms likely to be reported by callers about people in the prodromal stages of illness caused by the deliberate release of a biological or chemical agent. In monitoring these data, we also hoped to be able to detect community outbreaks of more common infectious diseases.  2003: Initial challenges included convincing people of the worth of syndrome/clinically based surveillance rather than microbiologically based surveillance (considered the ‘gold standard’ for surveillance). |
| **‘The growth phase’: 2006 to 2011** |
| 2006: First time analyses used to support heatwave response: first qualitative evaluation of the *usefulness* of primary care surveillance outputs for stakeholders  2009: Part of a European bid to assess and develop guidelines for Syndromic Surveillance Systems in Europe  2010: Increasing focus on mass gatherings with the upcoming London 2012 Olympic and Paralympic Games  2010: Requests for uses for environmental incidents such as the impact of the Icelandic ash plume - only sources of real-time health data. |
| **‘Mainstream’: 2012 to 2018** |
| 2014: Cold weather indicators defined and demonstrated to be useful in the identification and monitoring of the impact of extreme cold weather on health.  2015: Considered an ‘All Hazard’ service  2016: Increasing requests in support of infection and environmental incidents |

**Table S2.** Examples of the important developments in the **team and systems** of the UK Health Security Agency syndromic surveillance service (1998 to 2018).

| **‘The beginnings’: 1998 to 2005** |
| --- |
| 1999: Received a daily fax of 3 lines including NHSD ‘cold/flu’ calls from the West Midlands site only  1999: First ‘bulletin’ produced based on NHS Direct data only.  2001: First publication called ‘syndromic’ based on the pilot work: Can calls to NHS Direct be used for syndromic surveillance? Describing the pilot at three NHS Direct sites.  2003: Linked to obtain daily GP data for surveillance.  2004: First paper suggesting the use for early warning: ‘Early warning and NHS Direct: a role in community surveillance? |
| **‘The growth phase’: 2006 to 2011** |
| 2008: Now called the ‘Real-time Syndromic surveillance team’  2010: Developing the out of hours system planned and driven by the upcoming Olympic and Paralympic Games.  2011: Scientist appointed to develop the Emergency Department Surveillance System (EDSSS) |
| **‘Mainstream’: 2012 to 2018** |
| 2012: 22 ED sites reporting and 80% Out of hours coverage.  2013: Developed new statistical methodologies to enable automated ‘alarms’.  2013: NHS 111 was introduced and thus the NHS Direct system was discontinued, and the new NHS 111 system rebuilt.  2014: Co-lead theme on ‘Enhancing Syndromic Surveillance for early detection and assessing the extent of disease’ in the National Institute for Health and Care Research Health Protection Research Unit for Emergency Preparedness and Response (research grant). GP syndromic surveillance schemes had a joint coverage of 30 million population  2015: Pilot of an ambulance syndromic surveillance system |

**Table S3.** Examples of the **main outputs and uses** of the UK Health Security Agency syndromic surveillance service (1998 to 2018).

| **‘The beginnings’: 1998 to 2005** |
| --- |
| 2004: detected a significant rise in NHS Direct ‘cold/flu’ calls. This surveillance ‘signal’ heralded a rise in influenza circulating in the community.  2005: Provided support to the ‘Buncefield Fire Incident’- key event showing usefulness in an incident needing real time intelligence on presenting symptoms. |
| **‘The growth phase’: 2006 to 2011** |
| 2007: National rise in NHS Direct ‘difficulty breathing’ calls about the under 1 yr age group pre-dated reporting of, and then supported evidence of an earlier than expected rise in respiratory syncytial virus in England and Wales.  2007: Provided real time data and interpretation for flooding response.  2009: Provided regular reports during the H1N1 ‘swine flu’ pandemic, used as part of the evidence of established community spread. |
| **‘Mainstream’: 2012 to 2018** |
| 2012: Provided real time monitoring for three months during the London 2012 Olympic and Paralympic Games.  2018: The ‘Beast from the East’ winter storm heralded a significant spell of snow and low temperatures in the UK with clear impacts upon health and the health service usage observed. |
